# Supplementary material for: The interactome of intact mitochondria by cross-linking mass spectrometry provides evidence for coexisting respiratory supercomplexes
Source: Mol Cell Proteomics. 2017 Dec 8;17(2):216–32. doi: 10.1074/mcp.RA117.000470 (PMC5795388; doi:10.1074/mcp.RA117.000470)
Supplement: Supplemental Data [file supp_RA117.000470_133922_0_supp_23978_fzffwf.zip › spectra_annotation/mito_spectra_annotation/288-1-9-1-3-1.pdf]

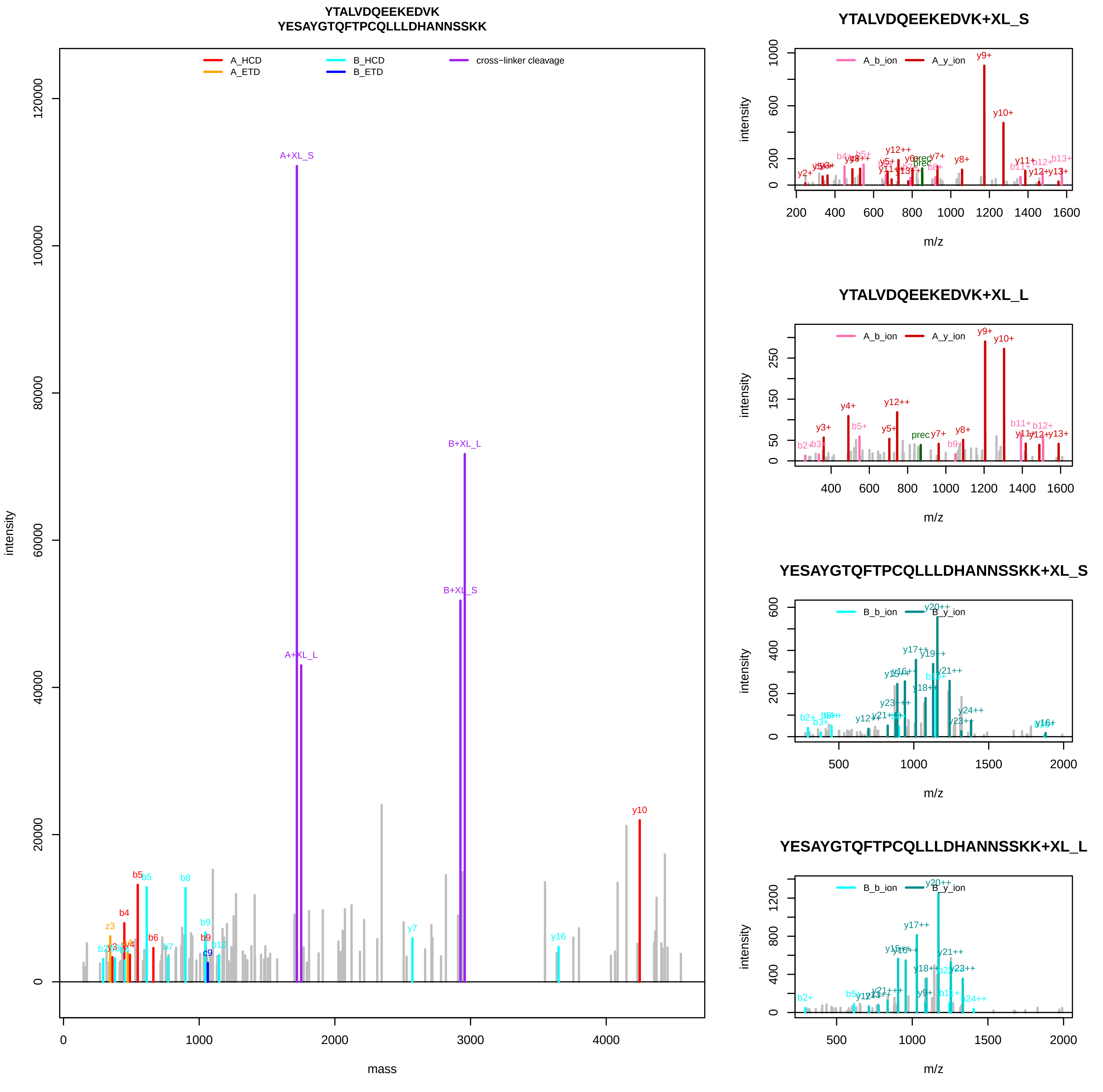
YTALVDQEEKEDVK
YESAYGTQFTPCQLLLDHANNSSKK
A_HCD
A_ETD
B_HCD
B_ETD
cross−linker cleavage
A+XL_S
A+XL_L
B+XL_L
B+XL_S
y10
b5
b4
z3
b6
b9
c9
b8
b7
b10
y7
y16
intensity
mass
YTALVDQEEKEDVK+XL_S
YTALVDQEEKEDVK+XL_L
YESAYGTQFTPCQLLLDHANNSSKK+XL_S
YESAYGTQFTPCQLLLDHANNSSKK+XL_L
A_b_ion
A_y_ion
B_b_ion
B_y_ion
y9+
y10+
y12++
y8+
y7+
prec
y4+
y3+
y5+
b5+
b11+
b12+
b13+
y11+
y12+
y13+
y20++
y17++
y19++
y21++
y18++
y24++
y23++
y16+
m/z
